# Local disease concepts relevant to the design of a community-based surveillance program for influenza in rural Guatemala

**DOI:** 10.1186/s12939-016-0359-z

**Published:** 2016-04-23

**Authors:** Alejandro Cerón, Maria Renee Ortiz, Danilo Álvarez, Guy H. Palmer, Celia Cordón-Rosales

**Affiliations:** Center for Health Studies, Universidad del Valle de Guatemala, Guatemala City, Guatemala; Department of Anthropology, University of Denver, Sturm Hall Room 131, 2000 E. Asbury Ave, Denver, CO 80208 USA; Paul G. Allen School for Global Animal Health, Washington State University, Pullman, WA USA

**Keywords:** Surveillance, Epidemiologic Surveillance, Community Participation, Influenza in humans, Influenza in birds, Zoonosis, Guatemala

## Abstract

**Background:**

Early detection of emergent influenza strains is a global health priority. However, maintaining active surveillance is economically and logistically challenging. While community-based surveillance is an attractive alternative, design and operation of an effective epidemiological surveillance program requires community engagement that can be linked to public health reporting and response. We report the results of a study in rural Guatemalan communities aimed at identifying opportunities for and barriers to community engagement in disease surveillance.

**Methods:**

Using an ethnographic approach followed by a descriptive cross-sectional survey, we documented local terms and ideas about animal illnesses, including the possibility of animal-human transmission.

**Results:**

The community perceived disease causation principally in terms of changes in the physical environment and weather and categorized illnesses using local terminology based on observable clinical signs. Knowledge about prevention and treatment was derived predominantly from local networks of family and friends without evidence of professionally-based knowledge being regularly introduced into the community.

**Conclusions:**

Bridging the divide between professional and community-based descriptive disease terminology, incorporating animal and human health responsiveness to common illnesses, and providing professional knowledge into the community-based networks were identified as addressable challenges to effective implementation of community-based surveillance.

## Background

Early detection of emergent influenza strains is a global health priority. Environments where wildlife is in close contact with domestic animals and humans are considered hotspots for the emergence of potentially pathogenic influenza strains [1–5]. The 2009 H1N1 virus responsible for the most recent pandemic contained genetic material derived from both avian and swine viruses and zoonotic transmission of the highly pathogenic avian H5N1 to humans resulted in an abnormally high case fatality rate [6, 7]. Notably, the H5N1 global spread was linked to movement of birds, both domestic poultry and migratory waterfowl [8–11]. Correspondingly, an increase in surveillance, especially in more vulnerable countries and populations where multiple animal species interact in close proximity to humans has been called for and, to varying degrees of success, implemented [12–14]. However maintaining active surveillance, especially in low income countries and communities where poultry and livestock are predominately raised in the absence of biosecurity and in close proximity to households, is both economically and logistically challenging given other pressing health and non-health priorities. An alternative approach is a community-based surveillance program in which the community itself is responsible for detecting changes in disease patterns in their livestock and poultry, understanding the risk of zoonotic transmission, and empowered to alert regional and national health authorities [15–17].

Community participation is essential for the design of efficient epidemiological surveillance programs. The systematic use of research methods applied to epidemiological programs facilitates the documentation of community understandings of disease and options for disease control. This type of research emphasizes community involvement in defining and prioritizing health-related problems, with the goal of improving detection, reporting, and response [18–20]. Participatory epidemiology [21] has been used to address zoonotic disease emergence, transmission, and control [22–28], including for avian influenza [16, 29–31]. As part of this, understanding how a community conceptualizes disease in their animals, assesses prevalence and categorizes specific diseases and patterns, and seeks both animal and human health guidance and treatment are essential.

Communities along Guatemala’s southern Pacific coast represent populations that are socioeconomically and epidemiologically vulnerable. Candelaria and Monterrico are the two main towns in the coast of the municipality of Taxisco, where 66 % of residents live in poverty and 18 % in extreme poverty [32], 44 % do not have access to safe sources of water or sanitary services [33], 25 % have no formal education, and 60 % only have some primary education [33, 34]. Predominately rural, families raise domestic animals for household consumption or as an income source. Poultry (predominately chickens and ducks) and swine are free ranging with frequent interactions among these species and with family members. Even if maintained with informal enclosures, poultry and pigs are closely located to the house and linked to the household through free-ranging dogs and cats and a contaminated environment. These free range and high contact rate conditions provide the opportunity to introduce new pathogens or pathogen strains into different animal hosts and humans that would not occur in other ecosystems where potential hosts are physically separated. Specifically free ranging household swine may have a critical role as they have frequently served as intermediate hosts for genetic reassortment of avian influenza viruses, resulting in strains transmissible to and pathogenic for humans [1, 3]. In addition to the role of free ranging household livestock, the Taxisco coast is a major overwintering zone for migratory aquatic birds: two natural reserves, Monterrico (2,800 ha) and Hawaii (3,657 ha) are large mangrove wetlands [35, 36]. Due to the close and frequent interactions between aquatic migratory fowl, domestic poultry and swine, and humans, these communities are potential hot spots for emergence and spread of influenza.

The Centro de Estudios en Salud de la Universidad del Valle de Guatemala, in collaboration with the U.S. Centers for Disease Control and Prevention, has conducted active surveillance for influenza in Taxisco, focused on these two communities directly bordering the Pacific Ocean, Monterrico and Candelaria [35]. Concomitantly, we initiated a study to identify opportunities for and barriers to community engagement in sustainable community-based surveillance. Using an ethnographic approach followed by a descriptive cross-sectional survey, we documented the characteristics of the main animal illnesses as defined by the community, as well as their signs, perceived causes, and consequent health-seeking behaviors. We report the findings of the study and present them in context of how community-based understanding of disease can be linked to improved public health reporting.

## Methods

### Research design

The study consisted of two phases. The first phase was exploratory and aimed at identifying the repertoire of local terms, concepts and practices relevant to animal health. The ethnographic approach used in this phase included informal interviews and observations, followed by qualitative analysis [37, 38]. The second phase was descriptive and aimed at documenting the extent to which the local terms, concepts and practices identified in the first phase were used by a representative sample of households. This phase used a cross-sectional survey and descriptive statistics [38]. Study design, data collection, and data analysis were all conducted in Spanish, the local language in the two villages. All authors and a research assistant are native speakers of Spanish, with the exception of GP, who is also fluent.

### Population and sample

The study was carried out in two villages, Monterrico and Candelaria, surrounding the Chiquimulilla Channel wetlands in the Pacific shore of Guatemala. This region was selected because it is an overwintering destination for migratory birds and household livestock include pigs, ducks, and chickens. There are twelve villages in this region, where domestic animals are in contact with the migratory fowl. The two selected villages are the ones with health posts staffed by the Ministry of Human Health, therefore considered to have greater potential for the future implementation of an epidemiological surveillance pilot program.

The ethnographic portion of the study used purposive sampling for selecting individuals with knowledge about animal illnesses and care (i.e. animal owners, healers), as well as places where animal care was delivered (i.e. backyards, kitchens). The cross-sectional survey was conducted in a sample of 90 households (42 in Monterrico and 48 in Candelaria) previously selected to randomly sample swine and duck populations as part of an active surveillance program. Monterrico and Candelaria have a total combined population of 961 inhabitants, and a total of 341 households according to a February 2013 census.

### Data collection

An initial phase of ethnographic research was conducted between February and April 2013. This phase consisted of observations and informal interviews conducted by a single interviewer (MRO) oriented by an observation and interview guide [37] aimed at identifying the local terms for household animal illnesses, as well as understanding the local beliefs and practices related to each of those illnesses as associated with care seeking behaviors. Species of interest for this ethnographic phase were chickens, ducks, and swine. Data from observations and informal interviews were documented in notebooks by the interviewer and transcribed into Microsoft Word. MRO and AC cross-checked transcriptions for accuracy and qualitatively analyzed them to identify relevant local terms and concepts..

A subsequent phase of cross-sectional survey was conducted in June 2013. The questionnaire design was informed by the findings of the ethnographic phase, and one questionnaire was administered per household. The questionnaire consisted of close-ended questions organized in six sections. An initial section was aimed at establishing prevalence of illness in the previous 15 days. If illness was reported, subsequent sections investigated name of illness, signs, perceived causes, treatments, and help sought by animal caregivers. Species of interest for this survey were chickens, ducks and swine. The questionnaire was created as an electronic form installed in a tablet computer, checked for accuracy by a field supervisor, and uploaded to a database that generated an Excel spreadsheet used for analysis. The questionnaire was validated by interviewing 10 households with the aim of detecting comprehension problems and to assess if the questions responded to the research aims.

### Data analysis

For the ethnographic phase, MRO and AC analyzed the original field notes and synthesized the findings into profile matrices for each of the reported illnesses. These matrices allowed organization of the information according to signs, perceived causes, treatments, and people/places sought for help. Matrices identified knowledge gaps to be addressed through additional interviews, and provided means for better understanding each of the illnesses as well as making comparisons between illnesses. Survey data displayed in a Microsoft Excel spreadsheet were analyzed using the pivot tables feature for generating tables of frequencies/percentages and cross tabulation analysis [38].

### Ethics, consent and permissions

The study protocol was approved by the Universidad del Valle de Guatemala’s Ethical Review Committee (protocol number 079-04-2013). All participants gave written informed consent. To preserve participants’ anonymity, neither the fieldnotes nor the survey documented household or individual identifiers.

## Results

### Animal ownership

A total of 55 informal interviews and 30 observations were documented through the ethnographic phase of the study, and a total of 90 households took part in the cross-sectional survey, with an average of five inhabitants per household (range 1–15). Ownership of animals, transportation assets, and education levels are provided in Table 1.

**Table 1:** Household characteristics (*n* = 90)

		Frequency	Percentage
Animal ownership	Chickens	62	69
	Ducks	17	19
	Swine	8	9
Transportation assets	Pickup truck	8	9
	Car	13	14
	Motorcycle	24	27
	Bicycle	67	74
Level of education (head of household)	Illiterate	16	18
	Primary school	54	60
	Secondary school	9	10
	Beyond secondary school	11	12

### Described illnesses, perceived causes, and treatments as identified by ethnographic research

Illnesses of chickens, ducks, and swine were specifically described (Table 2). Chicken illnesses include two with predominantly respiratory signs, (*coriza* and *soco*, the latter of which was also described as *pujo* or *moco*); two with gastrointestinal signs (*accidente por abajo* and *cólera*), two with problems of locomotion (*polio* or *apolio* and *calambres*), one with cutaneous signs (*viruela* or *buba*), and one with non-specific signs (*accidente por arriba*). Participants reported that common illness signs include sadness (*tristeza*), droopy beak (*pico caído*), inappetence (*no come*), and loss of feathers (*plumas caídas*). Illnesses are predominantly associated with changes in weather or climatic conditions, as well as with the presence of dust or dead animals. Treatments vary according to the predominant signs, with most illnesses treated with antibiotics and over-the-counter medications, some illnesses treated with local plants, and skin illnesses treated with topical procedures. Medications were procured primarily in local pharmacies and supply stores.

**Table 2:** Names and explanatory models for local illnesses in chickens, ducks, and swine

		Illness name^a^	System Affected	Signs	Causes	Treatments	Source of health care
Chicken	1.	Accidente por abajo	Gastrointestinal	Green diarrhea, inappetence, droopy wings,	Too much dust in the summer, too much rain in the winter. Unburied animals.	Water with batteries, water with coins, medicinal plants, tetracycline	Home care, local stores, local pharmacies.
	2.	Accidente por arriba	Respiratory	Lethargy, inappetence, droopy wings.	Too much dust in the summer, too much rain in the winter. Unburied animals.	No treatment. If treated, animal dies faster.	Home care.
	3.	Apolio/polio	Neuromuscular	Crooked legs, cannot walk.	Unspecified disease targets legs	Medicinal plants, codeine	Home care, local stores, local pharmacies.
	4.	Calambres	Neuromuscular	Lethargy, feather loss, legs ache.	Warm weather.	Medicinal plants	Home-care
	5.	Cólera	Gastrointestinal	Diarrhea, lethargy, inappetence.	Weather changes.	Medicinal plants, loperamide	Home-care, local stores, local pharmacies.
	6.	Coriza	Respiratory	Swollen face, dyspnea	Weather changes, dust.	Tetracycline, amoxicillin, naproxen	Home-care, local stores, local pharmacies.
	7.	Soco/pujo/moco	Respiratory disease	Nasal discharge, lethargy, inappetence	Weather changes.	Tetracycline, unspecified eye drops, other antibiotics, oil-soaked feather.	Local stores, local pharmacies, home-care.
	8.	Viruela/buba	Systemic/cutaneous	Hives, skin rash, blindness, fallen feathers	Wind carries microbes, mosquito bite, crescent moon.	Topical treatments involving heat (with a nail or charcoal) and iodine, chloride, or nail polish.	Home-care.
Duck	1.	Accidente	Systemic	Lethargy, inappetence, green diarrhea, hot ears	Too much dust in the summer, too much rain in the winter. Unburied animals.	No treatment. If treated, animal dies faster.	Home care.
	2.	Mal de ojo	Systemic	Lethargy, green diarrhea	Drunken men, sweating people	Red nail polish in the neck, medicinal plants, isolation.	Home-care, traditional healers.
	3.	Soco/moquillo	Respiratory disease	Nasal discharge, lethargy, inappetence	Weather changes.	Tetracycline, unspecified eye drops, other antibiotics, oil-soaked feather.	Local stores, local pharmacies, home-care.
	4.	Buba	Cutaneous	Hives, skin rash, blindness, fallen feathers	Weather changes.	Tetracycline, unspecified eye drops, other antibiotics, oil-soaked feather.	Local stores, local pharmacies, home-care.
	5.	Calambres	Neuromuscular	Lethargy, feather loss, legs ache.	Warm weather.	Medicinal plants	Home-care
	6.	Coriza	Respiratory	Swollen face, dyspnea	Weather changes, dust.	Tetracycline, amoxicillin, naproxen	Home-care, local stores, local pharmacies.
	7.	Accidente por abajo	Gastrointestinal	Green diarrhea, inappetence, droopy wings,	Too much dust in the summer, too much rain in the winter. Unburied animals.	Water with batteries, water with coins, medicinal plants, tetracycline	Home care, local stores, local pharmacies.
	8.	Apolio/polio	Neuromuscular	Crooked legs, cannot walk.	Unspecified disease targets legs	Medicinal plants, codeine	Home care, local stores, local pharmacies.
Swine	1.	Empacho	Gastrointestinal	Lethargy, inappetence hair loss	Wind, weather changes, dust, lack of vaccination.	Several antibiotics	Local pharmacies, home-care.
	2.	Mal de ojo	Systemic	Lethargy, green diarrhea	Drunken men, sweating people	Medicinal plants, isolation.	Home-care, traditional healers.
	3.	Parásitos	Gastrointestinal	Diarrhea, lethargy, inappetence	Lack of vaccination, eating garbage.	Lard mixed with panela (unrefined sugar).	Home-care
	4.	Rasquiña/jiote	Cutaneous	Alopecia, dermatitis, lethargy, inappetence	Contaminated water, lack of vaccination.	Vitamins, anti-parasite medication.	Local pharmacies.

^a^Illness names respect local terms

Illnesses in ducks are described very similar to those of chickens (Table 2), with the exception of the *mal de ojo* (evil eye), which is described in very similar terms as the syndrome amply described in humans [39]. Swine illnesses also include the *mal de ojo*, together with additional illnesses that present a combination of systemic, cutaneous, and gastrointestinal signs (Table 2). Perceived causes of illness, treatments, and help sought are similar to those described for chickens and ducks.

### Described illnesses, perceived causes, and treatments as identified by cross-sectional survey

The most common illness reported to have occurred in the 15 days prior to the day of the household survey was *soco*, found in 56 % of the households that raised chickens. *Accidente* was the most prevalent described illness in ducks (24 %) as was *jiote* in swine (50 %). Table 3 shows reported illness prevalence by species. Non-specific signs such as sadness (*tristeza*), inappetence (*no come*) and prostration (*echado*) were the most prevalent reported for each species, accounting for between 50 and 75 % of the households. Importantly, these general signs were the ones reported as having being first noticed by survey respondents. This pattern remains when analyzing respiratory illnesses (*soco, coriza*) and gastrointestinal illnesses (*colera, accidente por abajo*), where non-specific signs were reported as being noticed initially and more frequently than syndrome defining signs. In contrast, for diseases of the skin, feather or cutaneous lesions were the first identified signs. Reported signs of illness are shown in Table 4.

**Table 3:** Frequency of illness^a^

Species	Households reporting	Syndrome^b^	No. cases reported
Chicken	62	*Soco, pujo ó moco*	35
		*Viruela/buba*	22
		*Accidente*	16
		*Otras*	10
		*Accidente por arriba*	8
		*Coriza*	7
		*Accidente por abajo*	7
		*Apolio/polio*	5
		*Calambres*	5
		*Cólera*	3
Duck	17	*Accidente*	4
		*Otras*	4
		*Mal de ojo*	3
		*Soco o moquillo*	3
		*Buba*	2
		*Calambres*	2
		*Coriza*	1
		*Accidente por abajo*	1
		*Apolio/polio*	1
Swine	8	*Rasquiña o jiote*	4
		*Parásitos*	2
		*Empacho*	1
		*Mal de ojo*	1

^a^Recall during the previous 15 days

^b^Illness names respect the local use of terms in Spanish

**Table 4:** Households reporting signs of illness in the past 15 days, frequencies

Local term	Translation^a^	Chicken (*N* = 62)	Ducks (*n* = 17)	Swine (*n* = 8)
*Tristeza*	Sadness	45	10	2
*No come*	Not eating, lack of appetite	40	18	6
*Ronchas en la piel*	Skin rash	40	1	0
*Postración, Echado*	Prostration, recumbent	37	17	3
*Debilidad*	Weakness	37	7	1
*Cuesta respirar*	Difficulty to breath	37	6	0
*Mocos, flemas*	Nasal discharge	35	3	0
*Ojos hinchados*	Swollen eyes	32	3	1
*Alas caídas*	Droopy wings	31	7	--
*Fiebre*	Fever	30	4	2
*Cuesta caminar*	Difficulty to walk	29	9	1
*Deshidratación*	Dehydration	29	5	2
*Estornudos*	Sneezing	28	2	0
*Tos*	Coughing	26	2	1
*Secreciones oculares*	Eye discharge	25	2	1
*Ciego*	Blindness	24	7	0
*Plumas caídas/pelo caído*	Fallen feathers/hair	14	3	2
*Piel hinchada*	Swollen skin	8	1	1
--	Other signs	5	3	5

^a^Likely translation to English of the local terms used in Spanish

Perceived causes of illness were predominantly (60–80 % of reported illnesses) related to the physical environment and weather, such as temperatures warmer or cooler than normal, sudden changes in temperature, rain, and dust (Table 5). This pattern is even stronger for respiratory and gastrointestinal illnesses, where these climatic changes were perceived as the cause of up to 95 % of reported cases. Unburied dead animals were also reported as a perceived cause, but only in 20–35 % of cases. Skin illnesses were more often associated with non-weather related environmental contamination than were other illnesses but were also associated with dust due to changes in weather.

**Table 5:** Households reporting causes of illness in the past 15 days, frequencies

Reported Cause^a^	Translation	Chicken (*n* = 62)	Ducks (*n* = 17)	Swine (*n* = 8)
*Calor*	Hot weather	27	6	1
*Cambios de clima*	Weather changes	24	2	0
*Polvo*	Dust	23	5	1
*Verano*	Summer	22	3	2
*Lluvia*	Rain	18	2	0
*Virus*	Virus	16	5	1
*Aire*	Air	16	3	1
*Invierno*	Winter	16	2	0
*Mosquito*	Mosquito	16	2	0
*Animals muertos no enterrados*	Unburied dead animals	14	3	0
*Vendedores de animales*	Animal sellers	9	3	0
*Alimentos*	Food	4	0	2
*Sangre pesada, energía fuerte*	Ill-natured person, person with strong energy	1	3	0
*Persona sudada*	Sweaty person	0	4	0

^a^Causes names use the likely translation to English of the local terms used in Spanish

Diseases in both chickens and ducks were overwhelmingly treated with antibiotics obtained without prescription at local shops or pharmacies. Tetracycline and amoxicillin were the most commonly used antibiotics (Table 6). This was particularly true for respiratory and gastrointestinal illnesses such as *soco*, where 83 % of cases were treated with tetracycline. The fewer number of reported disease cases in swine limited assessment of specific treatment frequency, however both antibiotics and changing the animal’s diet were reported. The use of home remedies or medicinal plants was markedly less common. Treatment decisions were made in 90 % of cases by the household itself and in most of the remaining 10 % with the help of neighbors or relatives. Only two households reported having gone to a pharmacy or to a store specialized in animal products in the nearest city (Taxisco, 19 km from Monterrico, 22 km from Candelaria).

**Table 6:** Households reporting treatment of illness in the past 15 days

Treatment	Chickens (*n* = 62)	Ducks (*n* = 17)	Swine (*n* = 8)
Tetracycline	57	9	3
Other treatments	36	6	3
Amoxicillin	22	2	1
Battery water	9	1	0
Eye drops	6	1	0
Oil-soaked feather	6	0	0
Scrape the skin	5	1	0
Iodine	5	0	0
Massage, rubbing	4	3	0
Medicinal bath	2	1	0
Isolation	0	3	0
Red nail polish	0	1	0
Rubbing with medicinal plants	0	1	0
Avoid certain foods	0	0	1

## Discussion

Local terms and understandings of animal health problems pose challenges to the implementation of community-based surveillance programs because they highlight the knowledge and language gaps between institutional and community actors. The scientific underpinnings of infectious disease transmission and, especially, drivers for emergence of new zoonotic pathogens are unlikely to be well understood in communities with a low level of formal education unless translated into locally recognizable terms. This is reflected in the two rural communities involved in this study--over 75 % of the population lacks secondary or higher education. This is exacerbated by the very limited public health and animal health resources available to the communities. This gulf in scientific understanding of infectious disease concepts and the language used to express these concepts between the formal public health sector and the communities most likely to first encounter an emerging zoonotic pathogen is an impediment to implementing community-based surveillance. However, this impediment can be markedly reduced by building bridges between what the community observes and how these observations can be translated into actionable public health information. This way, the challenge posed by local understandings of animal health problems is transformed into an opportunity for public health practitioners implementing epidemiological surveillance.

### Early detection

The community members clearly associate disease with changes in seasons and local weather patterns. This association is compatible with patterns of certain infectious diseases, including seasonal influenza. This is similarly relevant to the spread of new influenza viruses associated with seasonal migrations of wild waterfowl. Furthermore, the community members are observant in reporting initial, more general signs of disease, which strengthens their ability to detect early events. Understanding the local terminology and perceptions of animal health problems can aid public health practitioners in bridging the gaps that make it difficult to implement community-based surveillance programs.

### Diagnosis

The public health community needs to understand diseases of epidemiologic priority in the context of endemic diseases as understood by local communities. Descriptive names may be linked to specific pathogens or, alternatively, be more generalized and encompass multiple pathogens unified only by initial presentation. Rabies for example, derived from the Latin for rage, has evolved from a syndromic description to represent a specific etiology that is commonly understood at both community and public health institutional levels. In contrast, “flu” denotes a specific etiology, influenza virus, at the public health level but may represent a variety of etiologies at the community level that have similar elements of clinical presentation. Providing diagnostic services focused on common illnesses in rural communities can define specific etiologies or sets of etiologies linked to the syndrome that would improve translation across educational divides, essentially focusing on education of public health professionals in the meaning of local terminologies rather than the reverse. By focusing diagnostic services on endemic diseases, the ability to detect a shift in disease signs, patterns, or affected species will be enhanced, a requirement for detection of a newly emergent pathogen.

### Treatment and prevention

Importantly, diagnosis of disease must be accompanied by action, whether vaccination, treatment or changes in animal husbandry, in order to provide an incentive for reporting in community-based surveillance. Decisions about whether or not to treat an ill animal and with what were heavily influenced by neighbors rather than by animal health professionals. This in itself is unsurprising and is not necessarily linked to either level of education or socioeconomic status. For example, even in wealthy countries with available maternal health care services, expectant and new mothers seek and act on advice from experienced mothers among family and friends. However, there is also a need and opportunity for evidence-based information to be introduced into those familial and social networks [40]. This also applies to introducing the concept of infectious agents as a cause of disease. Attribution of disease causation only to climatic events, clearly not under the control of individuals or the community, inhibits the implementation of effective preventive measures, such as vaccination and animal husbandry. Use of the community engagement initiated during this study offers the opportunity to provide this information, especially to leaders within the community, and therefore take advantage of the existing community structure for dissemination.

Limitations of this study include those known for cross-sectional studies, and reliance on self-reported knowledge and behaviors. Results are only representative of the two studied villages.

## Conclusions

Community-based surveillance of diseases of high epidemiologic priority is necessary and feasible. An estimated 60 % of new human infectious diseases emerge from animals and the role of animals, especially swine, chickens, ducks, and migratory waterfowl, is well established for emergence and spread of new influenza viruses [5]. Although many zoonotic pathogens emerge in rural areas with a scarcity of either animal or human health services, they remain undetected until either affecting large numbers of people or animals or spreading to urban areas where detection capacity and capability exists. Enhanced community-based surveillance provides the opportunity to enable earlier detection and response by community-based recognition of a change in disease pattern and prompt notification of public health authorities. Understanding how communities view and express disease concepts, and engaging with them on their endemic disease problems, provides the opportunity to link their continuous observations and local knowledge to effect enhanced surveillance. Areas for future research include those aimed at designing participatory surveillance systems and measuring their effectiveness in disease control.
